# A Deep Learning Approach for Automatic and Objective Grading of the Motor Impairment Severity in Parkinson’s Disease for Use in Tele-Assessments

**DOI:** 10.3390/s23219004

**Published:** 2023-11-06

**Authors:** Mehar Singh, Prithvi Prakash, Rachneet Kaur, Richard Sowers, James Robert Brašić, Manuel Enrique Hernandez

**Affiliations:** 1 Computer Science and Engineering Division, University of Michigan, Ann-Arbor, MI 48109, USA; mehars@umich.edu; 2 Department of Computer Science, University of Illinois Urbana-Champaign, Urbana, IL 61801, USA; 3 School of Information Sciences, University of Illinois Urbana-Champaign, Champaign, IL 61820, USA; prithvi6@illinois.edu; 4 Department of Industrial and Enterprise Systems Engineering, University of Illinois Urbana-Champaign, Urbana, IL 61801, USA; rk4@illinois.edu (R.K.); r-sowers@illinois.edu (R.S.); 5 Section of High Resolution Brain Positron Emission Tomography Imaging, Division of Nuclear Medicine and Molecular Imaging, Russell H. Morgan Department of Radiology and Radiological Sciences, The Johns Hopkins University School of Medicine, Baltimore, MD 21205, USA; 6 Department of Behavioral Health, New York City Health + Hospitals/Bellevue, 462 First Avenue, New York, NY 10016, USA; 7 Department of Psychiatry, New York University Grossman School of Medicine, New York University Langone Health, New York University, 550 First Avenue, New York, NY 10016, USA; 8 Neuroscience Program, Beckman Institute, College of Liberal Arts & Sciences, University of Illinois Urbana-Champaign, Urbana, IL 61801, USA; 9 Department of Biomedical and Translational Sciences, University of Illinois Urbana-Champaign, Urbana, IL 61801, USA; 10 Department of Kinesiology and Community Health, University of Illinois Urbana-Champaign, Urbana, IL 61801, USA

**Keywords:** artificial intelligence, bradykinesia, machine learning, movement disorders, multidisciplinary, postural instability, pronation, rigidity, supination, tremor, wearables

## Abstract

Wearable sensors provide a tool for at-home monitoring of motor impairment progression in neurological conditions such as Parkinson’s disease (PD). This study examined the ability of deep learning approaches to grade the motor impairment severity in a modified version of the Movement Disorders Society-sponsored revision of the Unified Parkinson’s Disease Rating Scale (MDS-UPDRS) using low-cost wearable sensors. We hypothesized that expanding training datasets with motion data from healthy older adults (HOAs) and initializing classifiers with weights learned from unsupervised pre-training would lead to an improvement in performance when classifying lower vs. higher motor impairment relative to a baseline deep learning model (XceptionTime). This study evaluated the change in classification performance after using expanded training datasets with HOAs and transferring weights from unsupervised pre-training compared to a baseline deep learning model (XceptionTime) using both upper extremity (finger tapping, hand movements, and pronation–supination movements of the hands) and lower extremity (toe tapping and leg agility) tasks consistent with the MDS-UPDRS. Overall, we found a 12.2% improvement in accuracy after expanding the training dataset and pre-training using max-vote inference on hand movement tasks. Moreover, we found that the classification performance improves for every task except toe tapping after the addition of HOA training data. These findings suggest that learning from HOA motion data can implicitly improve the representations of PD motion data for the purposes of motor impairment classification. Further, our results suggest that unsupervised pre-training can improve the performance of motor impairment classifiers without any additional annotated PD data, which may provide a viable solution for a widely deployable telemedicine solution.

## 1. Introduction

Parkinson’s disease (PD) is a prevalent neurodegenerative disorder that is characterized by motor symptoms including bradykinesia, rigidity, resting tremor, and postural instability [1,2], while there is no cure for PD, access to multidisciplinary medical care can greatly benefit people with PD [3]. However, clinical evaluations typically require limited-availability in-person appointments with a movement disorders specialist at limited locations, requiring significant travel time and hampering access [4]. Given the expected increase in the prevalence of PD expected in the population [5], there is a significant need for telemedicine solutions to improve access to healthcare for people with PD [6].

Traditional clinical evaluations of PD patients can often be time-consuming and inefficient for both patients and clinicians. While multiple rating scales have been proposed, the gold standard evaluation of motor impairment in people with PD is the Movement Disorder Society-sponsored revision of the Unified Parkinson’s Disease Rating Scale (MDS-UPDRS) [7]. The MDS-UPDRS motor subsection provides a qualitative assessment of bradykinesia, resting tremor, and postural instability symptoms, but not rigidity. However, there is a lack of automatic and objective evaluations of motor impairment for potential integration in telemedicine applications.

Telemedicine solutions may be well suited to evaluate people with PD, particularly in rural and underserved communities that usually lack access to healthcare [8,9]. Teleconsultations may provide an opportunity for the remote administration of neurological examinations, but subtle features such as bradykinesia may be difficult to capture using video alone [10]. Additionally, rigidity cannot be assessed without a physical examination, with hands-on manipulations of the joints of the patient by the examiner. While the COVID-19 pandemic fast tracked the deployment of telemedicine applications to address the needs of people with PD, there are still challenges related to inconsistent monitoring images, privacy, poor connectivity, and access to technology.

The use of machine learning with behavioral data has shown great promise in differentiating the pathological and physiological motor responses arising from PD [11,12,13] and several other neurological conditions [14,15] from healthy controls. The integration of low-cost wearable technology and deep learning approaches may provide a viable approach towards the development of robust and more widely deployable telemedicine solutions.

However, one of the major challenges with machine learning in telemedicine is the extensive amounts of labeled data required to train supervised learning models [16]. There exists a shortage of available datasets on motion data from PD patients in addition to the costliness and inefficiency of manually annotating data. As such, further development of unsupervised learning and transfer learning techniques is needed in telemedicine. Unsupervised learning is the process of learning from unlabeled data, while transfer learning is the process of using the knowledge acquired from one objective, perhaps with more abundant data, to an adjacent objective. However, it is important to establish specific approaches for a given application when using transfer learning principles [17].

In this study, we explored how transferring unsupervised pre-trained weights onto supervised models can improve the performance of motor impairment classification for PD patients. We experimented with models trained on datasets containing motion sequences from only PD patients as well as both PD patients and healthy older adults (HOAs). Firstly, we hypothesized that the inclusion of HOA motion sequences in training datasets would lead to performance improvements, since such sequences could act as a healthy control for anomaly detection. Secondly, we predicted that using unsupervised pre-trained weights to initialize our supervised classification models would improve the performance relative to random weight initialization (RandInit). We suspected that the unsupervised models from which we transferred weights would implicitly learn meaningful representations of motion sequences for the motor impairment classification objective.

Overall, we sought to determine if unsupervised learning, transfer learning, and the inclusion of HOA motion sequences in training datasets can improve motor impairment classification for PD patients without using any additional data from PD patients.

## 2. Materials and Methods

### 2.1. Protocol

We collected continuous motion data from the extremities of both PD and HOA participants using a custom-built, low-cost quantitative measurement system. It consisted of a 3-axis accelerometer (ADXL335) and an evaluation board (EVAL-ADXL335Z) with USB connectors [18]. We received ground-truth evaluation of movement impairment from an examiner certified in the administration of the Movement Disorder Society-sponsored revision of the Unified Parkinson’s Disease Rating Scale (MDS-UPDRS) [7,18].

For upper extremity movements (finger tapping (FT), hand movements (HMs), and pronation–supination movement of the hands (PS)), repetitive movements were recorded at 80 Hz using two accelerometers placed at:

1. The dorsal surface of the second (middle) phalanx of each index finger;
2. The dorsum of each arm, midway between the radius and the ulna and two inches proximal to each wrist joint.

For lower extremity movements (e.g., toe tapping (TT) and leg agility (LA)), repetitive movements were recorded at 80 Hz using two accelerometers placed at:

1. The anterior surface of each tibia, two inches proximal to the medial malleolus;
2. The dorsal surface of the proximal phalanx of each big toe.

**Figure 1:**
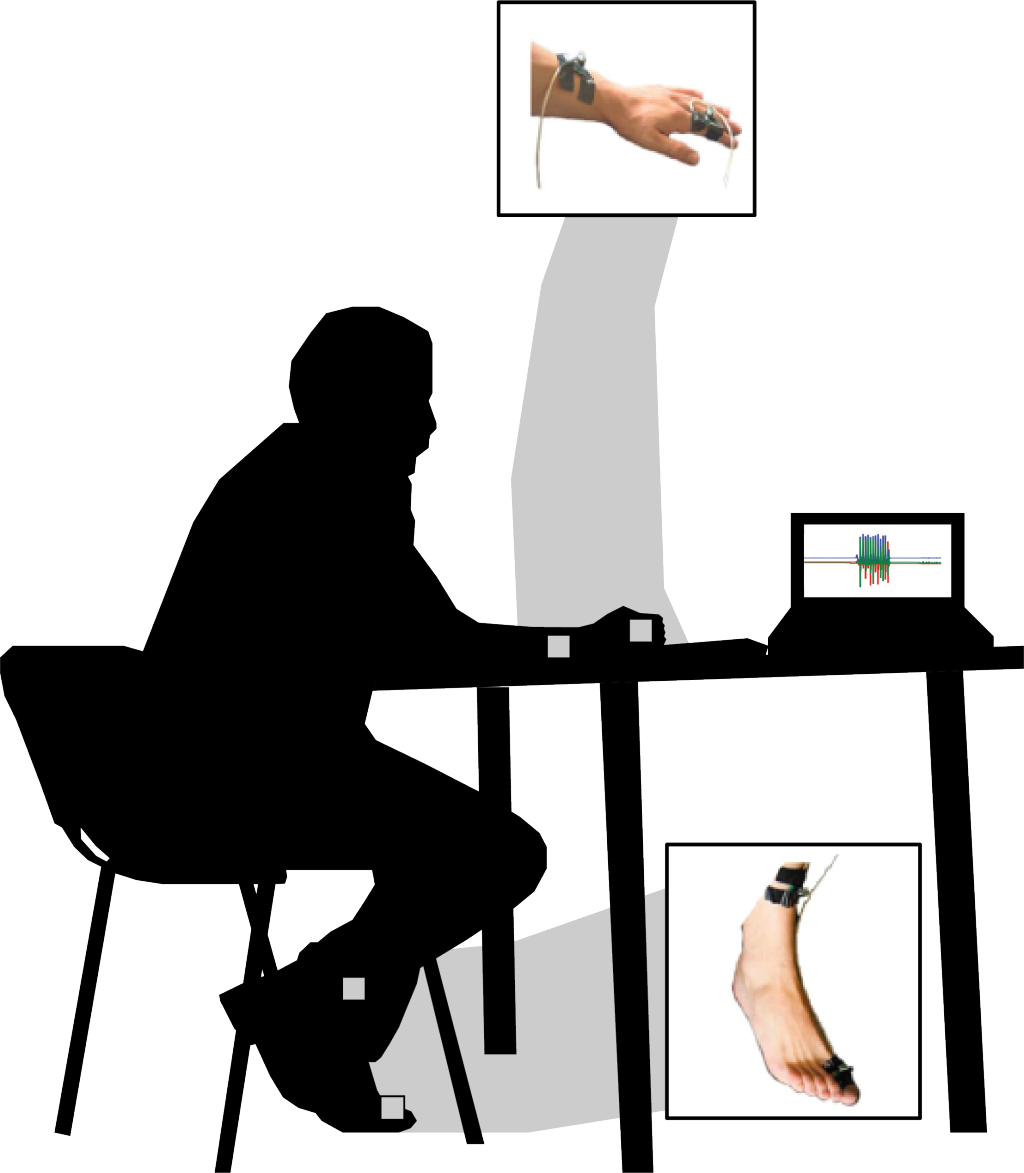
The setup for sensor placement for upper extremity and lower extremity data collection. Images of the hand and the foot are reproduced with permission [18].

Participants returned after a week or more for repeat testing. Figure 1 illustrates the sensor placement setup used for data collection.

### 2.2. Participants

We used an open-source dataset [19] that recorded 28 participants composed of 20 individuals with PD (14 males, mean age = 67 ± 10 years) and 8 HOAs (five males, mean age = 64 ± 7 years). Additionally, 19 of these participants had a retest session. This study utilized 16 recorded sessions from HOAs and 32 recorded sessions from individuals with PD. The data were collected in accordance with the Code of Ethics of the World Medical Association (Declaration of Helsinki) and the protocol was approved by the Johns Hopkins Institutional Review Board. Written informed consent was obtained from all participants utilizing a protocol (Protocol Number: IRB00110 166 and Initial Approval Date: 22 September 2016) approved by the Johns Hopkins Medical Institutions Institutional Review Board, Baltimore, MD, USA.

### 2.3. Deep Learning Hardware and Software Tools

All of our experiments were performed on an Apple M2 Max machine Ventura 13.4 using a 12-core CPU. We trained our models using tsai v0.3.6 [20], an open-source deep learning tool built on top of fastai [21] and PyTorch [22], in Python v3.9.17.

### 2.4. Data Preprocessing

For each participant’s session in which a task (FT, HM, PS, TT, LA) was performed, the resultant acceleration of the sensor *i* at timestep *j* was calculated as:

$$A_{ij}=\sqrt{a_{xij}^{2}+a_{yij}^{2}+a_{zij}^{2}}$$

where $a_{x},a_{y},a_{z}$ represent acceleration in the x-axis, y-axis, and z-axis. We then defined the acceleration sequence as:

$$S=\left[\begin{array}{cc} A_{1,1} & A_{2,1}\\ A_{1,2} & A_{2,2}\\ \vdots & \vdots \\ A_{1,N} & A_{2,N} \end{array}\right]$$

where the length *N* of the recording was arbitrary, and two accelerometers were used. The derivative of the resultant of each accelerometer, or jerk, was calculated. Further, the root mean square was taken over the time series sequences using a 1 s window. Since the length *N* of each time series sequence was arbitrary, overlapping snippets of five seconds in duration (i.e., 400 data points) from the recordings were segmented out. Each segmented sequence was standardized at an individual sample level (mean and standard deviation were calculated for each segmented sequence). Additionally, each of these segmented sequences shared a motor impairment severity label associated with the movement of the participant for the given task. These labels, originally scaled in the range of 0 to 4, were aggregated to either low (0/1) or high (3/4) levels of motor impairment. We ignored all sequences labelled as severity level 2.

### 2.5. Training, Validation, and Test Sets

**Figure 2:**
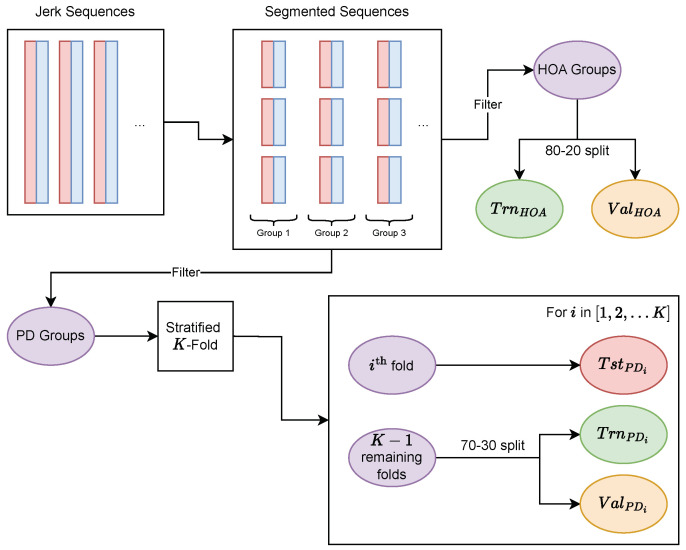
The divisions of PD and HOA sequences used to create training, validation, and test groups. Abbreviations: HOA = healthy older adult; PD = Parkinson’s disease.

The first step was to divide our sequences and labels into training, validation, and test sets, as Figure 2 displays. For each task, we selected the corresponding segmented sequences. All segmented sequences that originally belonged to the same original sequence were placed in the same group. In doing so, we prevented segmented sequences from the same test session from crossing over from training or validation sets to the test sets, which could artificially boost the model performance. We then filtered these groups into a set of groups for HOA participants and a set of groups for PD participants. The HOA groups were divided into a training set $Trn_{HOA}$ and a validation set $Val_{HOA}$ with an 80-20% random split.

The set of PD groups went through a stratified *K*-fold, which divided the set of groups into *K* equally sized folds while maintaining the associated label distribution in each fold. We used K=5 in our implementation. We iterated from i=1 to *K* and set the test set $Tst_{PD_{i}}$ to the $i^{\mathrm{th}}$ fold. The training set $Trn_{PD_{i}}$ and the validation set $Val_{PD_{i}}$ were set by a 70-30% random split of the remaining K−1 folds. The stratified *K*-fold guarantees that every segmented PD sequence is used at least once to evaluate the model’s performance via the test set. As such, it mitigates some of the bias surrounding model performance estimations that is caused by the arbitrary selection of training, validation, and test datasets.

### 2.6. XceptionTime Architecture Selection

**Figure 3:**
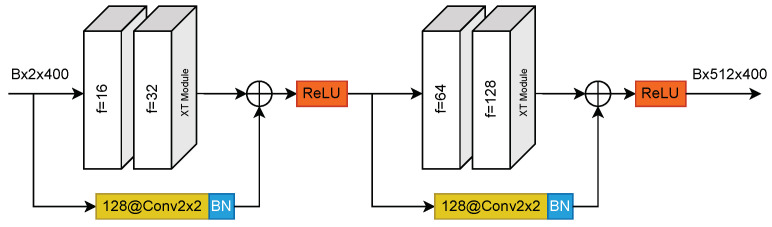
XceptionTime (XT) block architecture. Abbreviation: B = batch size; BN = batch normalization; f = $\frac{\mathrm{the}\;\mathrm{number}\;\mathrm{of}\;\mathrm{output}\;\mathrm{channels}}{4}$.

XceptionTime [23] is a convolutional neural network architecture for time-series data, as shown in Figure 3. We used XceptionTime for in deep learning model in this paper since it has empirically been shown to perform well on motor impairment classification for individuals with PD [24]. Our experiment can be reproduced with other neural network architectures. The architecture used for the unsupervised models must be the same as the architectures used for the supervised models since our method involves transferring weights from the unsupervised models to the supervised models, which is only possible if their architectures are identical.

### 2.7. Unsupervised Learning: Procedure

Recent work [25] has demonstrated the ability of unsupervised neural networks to implicitly learn deep representations of multivariate time-series inputs when trained to predict masked portions. The pre-trained weights from such unsupervised models have been shown to offer performance benefits when transferred to supervised models for classification, even when the training datasets are small. We employed this method by independently masking a proportion *r* of the input with zeros. The masking was applied such that:

1. The length of each masked segment was drawn from a geometric distribution with a mean of $l_{m}$.
2. Each succeeding unmasked segment had a mean length $l_{u}=l_{m}\frac{1-r}{r}$.

**Figure 4:**
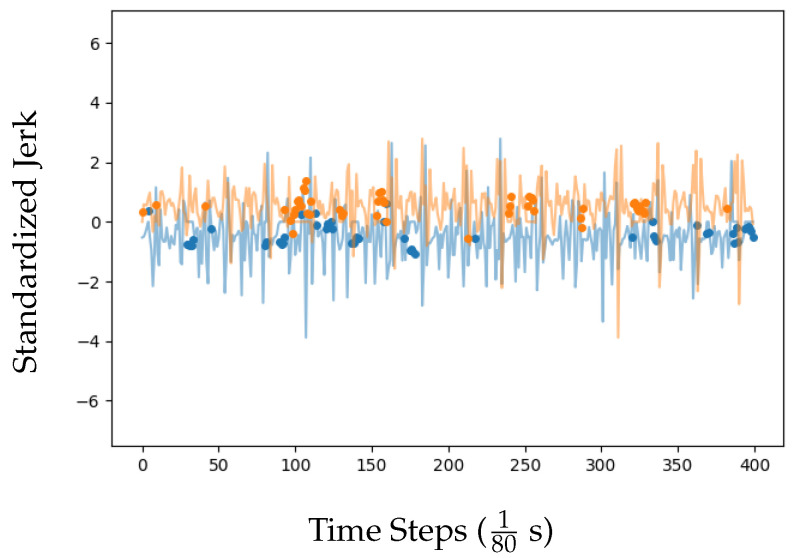
Example predictions, made by one of our unsupervised XceptionTime models, of masked portions of an input motion sequence from an HOA participant. The two colors correspond to the two sensors that make up the multivariate time-series sequence. Abbreviations: HOA = healthy older adult.

Our procedure used the hyper-parameters r=0.15 and $l_{m}=3$. We trained unsupervised XceptionTime models for 200 epochs with a maximum learning rate of $10^{-3}$. They were optimized using the mean squared error loss function:

$$L_{MSE}=\frac{1}{N}\sum_{i=1}^{N}{\left(y_{i}-{\hat{y}}_{i}\right)}^{2}$$

where $y_{i}$ is the *i*th masked input, ${\hat{y}}_{i}$ is the predicted *i*th masked input, and *N* is the total number of masked inputs. Figure 4 displays an example of the masked predictions made by one of our unsupervised XceptionTime models.

### 2.8. Unsupervised Learning: Pre-Trained Weight Generation

**Figure 5:**
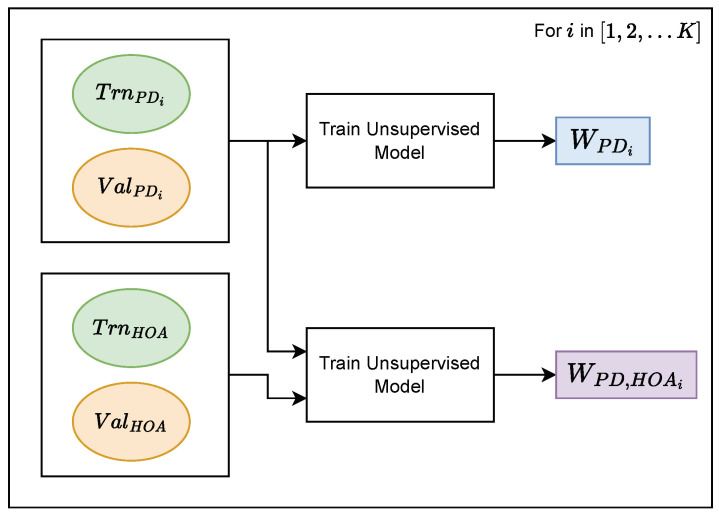
The combination of training and validation sets used to train unsupervised learning models and generate pre-trained weights. Abbreviations: HOA = healthy older adult; PD = Parkinson’s disease; *W* = weights.

Using the specifications detailed in Section 2.7, we trained unsupervised XceptionTime models for each task and saved their weights. A workflow of our unsupervised learning procedure can be found in Figure 5. For each task, we retrieved $Trn_{HOA}$ and $Val_{HOA}$. Then, we iterated from i=1 to *K* and retrieved $Trn_{PD_{i}}$ and $Val_{PD_{i}}$. We used $Trn_{PD_{i}}$ and $Val_{PD_{i}}$ to train and validate an unsupervised learning model, whose learned weights we saved as $W_{PD_{i}}$. Then, we trained another unsupervised learning model using the sequences from both $Trn_{PD_{i}}$ and $Trn_{HOA}$. This model was validated from both $Val_{PD_{i}}$ and $Val_{HOA}$. The learned weights of this model were saved as $W_{PD,HOA_{i}}$.

### 2.9. Supervised Learning Procedure

For each task, we iterated from i=1 to *K* and trained 10 different supervised XceptionTime models. Of the ten models, five used $Trn_{PD_{i}}$ as the training dataset, $Val_{PD_{i}}$ as the validation dataset, and $Tst_{PD_{i}}$ as the test dataset to evaluate motor impairment classification performance. The other five used $Trn_{PD_{i}}\cup Trn_{HOA}$ as the training dataset, $Val_{PD_{i}}\cup Val_{HOA}$ as the validation dataset, and $Tst_{PD_{i}}$ as the test dataset. Note that every test set neglected HOA data to only record the model performance on classifying motor impairment for PD patients.

In each group of five supervised XceptionTime models, one model used random initialization for its weights. It underwent training for 50 epochs with a maximum learning rate of $10^{-3}$. Among the remaining four models, two were initialized with the pre-trained weights $W_{PD_{i}}$, while the other two were initialized with the pre-trained weights $W_{PD,HOA_{i}}$. For each pair of models using the same pre-trained weights for initialization, the first model had its head classification layers fine-tuned for 10 epochs. Subsequently, every layer was fine-tuned for an additional 50 epochs with a maximum learning rate of $10^{-3}$. This technique is referred to as “fine-tune last, then all” (FTL). In contrast, for the second model in each pair, every layer was fine-tuned for 50 epochs with a maximum learning rate of $10^{-3}$. This technique is referred to as “fine-tune all” (FTA).

All of the supervised XceptionTime models were optimized using the binary cross entropy loss function:

$$L_{BCE}=-\frac{1}{N}\sum_{i=1}^{N}\left(y_{i}\log\left(p_{i}\right)+\left(1-y_{i}\right)\log\left(1-p_{i}\right)\right)$$

where *N* is the number of training sequences, $y_{i}$ is the true label (motor impairment level), and $p_{i}$ is the predicted probability of high motor impairment.

We considered the supervised XceptionTime model trained with $Trn_{PD}$ and initialized with random weights in the baseline model, since it does not use pre-trained weights or HOA data. A summary of all the models created can be found in Table 1.

### 2.10. Model Inference

Recall that the original sequences were segmented into 400 time steps. We evaluated two forms of inference from our supervised XceptionTime models on test datasets containing PD sequences. The first was segmented inference, where a motor impairment prediction was made from a single segmented sequence. The other was max-vote inference, where the motor impairment prediction for an original sequence was based on the mode of the predictions from its segmented sequences. For each form of inference, we saved and averaged metrics including the accuracy and F1 score.

### 2.11. Aggregate Models

In addition to classifying motor impairment in individual motor tasks (PD, HM, FT, TT, LA), we also trained aggregate unsupervised and supervised XceptionTime models. To do this, we followed the same steps as described in the previous Section 2.5, Section 2.6, Section 2.7, Section 2.8 and Section 2.9 without filtering the segmented sequences for a particular task.

### 2.12. Evaluating Robustness

In real-world applications, factors including administrator inexperience, positioning, and calibration issues can make accelerometer sensor measurements unexpected and noisy. To analyze how our models’ robustness to perturbations is correlated with their training set ($Trn_{PD}$, $Trn_{PD}\cup Trn_{HOA}$) and weight initialization (RandInit, $W_{PD}$, $W_{PD,HOA}$), we added Gaussian noise to each segmented sequence in our test sets and re-evaluated the accuracies. The Gaussian noise was generated with mean μ=0 and standard deviation σ=0.05.

### 2.13. Quantifying Representational Similarities

**Figure 6:**
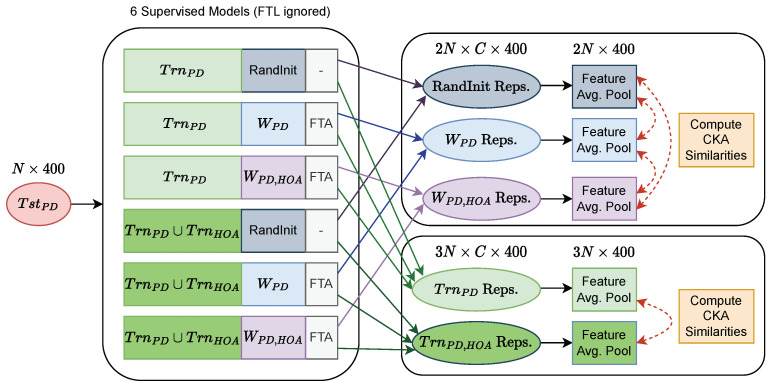
A workflow depicting the process of computing CKA similarity indexes for the representations among models with different initializations and training sets. Abbreviations: CKA = centered kernel alignment; FTA = fine-tune all; FTL = fine-tune last, then all; HOA = healthy older adult; PD = Parkinson’s disease; RandInit = random initialization; *W* = weights.

To understand how the internal behaviors of our supervised XceptionTime models for each task changed depending on their training set and initialization, we used centered kernel alignment (CKA) [26] to measure the feature similarity of the representations from convolutional layers between models with different training sets ($Trn_{PD}$, $Trn_{PD}\cup Trn_{HOA}$) and with different weight initializations (RandInit, $W_{PD}$, $W_{PD,HOA}$). CKA is a recently introduced similarity index that measures the similarity of deep neural network representations. Specifically, we evaluated the similarity of the representations from both the first and last XceptionTime modules (Figure 3) within the XceptionTime block. We omitted all models that used the FTL fine-tuning scheme in order to obtain the same amount of models initialized with RandInit, $W_{PD}$, and $W_{PD,HOA}$. Figure 6 displays how the representations were aggregated by model attributes to compute CKA similarities.

## 3. Results and Discussion

### 3.1. Best Performing Models

Table 2 and Table 3 show the best performing models in each task using segmented and max-vote inference, respectively. For segmented inference, we show that expanding the training dataset to include $Trn_{HOA}$, using pre-trained weights during initialization, or both yielded improvements in accuracy over the baseline for every task, as well as for aggregates. The same is reflected in max-vote inference, except for the TT task, in which the baseline model was tied for the highest accuracy.

Under max-vote inference, every model that showed at least 10% improvement over the baseline used $Trn_{PD}\cup Trn_{HOA}$ as the training set and $W_{PD}$ for weight initialization. Overall, the best performing models used $Trn_{PD}\cup Trn_{HOA}$ for four of five tasks (as well as for aggregates), and were initialized with $W_{PD}$ for four of five tasks. This provides some indication that HOA training data and pre-training can both boost accuracy for motor impairment classifiers. The model with the highest max-vote accuracy was trained on the HM task with $Trn_{PD}$ as its training set and was intialized with $W_{PD,HOA}$. It achieved an average accuracy of 92%, which was a 12.2% improvement over the baseline model for the HM task.

### 3.2. Benefits of HOA Training Data

Table 4 shows the average accuracy for our supervised XceptionTime models, grouped by their training set attribute ($Trn_{PD}$, $Trn_{PD}\cup Trn_{HOA}$) and their weight initialization attribute (RandInit, $W_{PD}$, $W_{PD,HOA}$). The models for three of the five tasks, as well as for aggregates, exhibited max-vote accuracy improvements when using $Trn_{PD}\cup Trn_{HOA}$ compared to just $Trn_{PD}$. The models for PS improved the most with the inclusion of HOA training data, by 5.9% on average. Our results show that, generally, HOA data should be considered as an addition to training datasets for motor impairment classification. For every task except TT (and including the aggregate), adding HOA data to the training set produced comparable or improved average accuracies.

We suspect that the addition of HOA training data improves the performance by making models more generalizable and robust. Recent work [27] has demonstrated that for machine learning models on medical tasks, adding more subtypes to training data increases the variability of the dataset, reduces overfitting, and improves model robustness. Our experiments reflect this as well. As Table 4 displays, the average accuracies of our supervised XceptionTime models reduced considerably with Gaussian noise applied to each segmented sequence. Despite this, when trained on $Trn_{PD}\cup Trn_{HOA}$ compared to just $Trn_{PD}$, the models for four of five tasks (with TT being the exception), as well as for the aggregate, exhibited both an improvement in the average max-vote accuracy and a reduction in the variance of model accuracy across all *K* trials. The models for FT improved the most, by 5.83% on average. The improvements that training with $Trn_{PD}\cup Trn_{HOA}$ provide for both the original and noisy motion sequences are equal [28], which highlights a correlation between better performing neural networks and neural networks that are more robust to perturbations.

### 3.3. Benefits of Pre-Training

As displayed by Table 4, the models for all five tasks exhibited max-vote accuracy improvements when initialized with pre-trained weights compared to random weights. While random initialization performed the best for the aggregate case, using either set of pre-trained weights ($W_{PD}$ or $W_{PD,HOA}$) led to comparable results that were only 0.8% less accurate on average. In contrast, the models for HM and LA improved the most with pre-trained weight initialization. Specifically, the models for HM saw an average improvement of 5.6% when initialized with $W_{PD,HOA}$ compared to random weights. The models for LA saw an average improvement of 6.1% when initialized with $W_{PD}$ compared to random weights. Weight initialization with $W_{PD,HOA}$ performed best for LA, while initialization with $W_{PD}$ performed best for PS, HM, FT, and TT.

Overall, we have demonstrated that the use of pre-trained weights, learned from masked unsupervised learning (Section 2.7), increases the motor impairment classification performance. The recent literature [29,30] attributes this fact, in part, to the increased generalization that pre-trained weights provide, as they have already learned the prevalent features from the source domain. Our findings are consistent with prior work, which shows that for small datasets, transferring and fine-tuning pre-trained weights for PD classification specifically can lead to a better performance compared to models initialized with random weights [31].

Pre-trained weights have also been shown to improve the robustness of machine learning models [32,33,34] for downstream tasks, including PD classification [35]. However, as Table 4 shows, we observed no significant performance improvements resulting from pre-trained weights compared to randomly initialized weights when Gaussian noise was applied to the motion sequences. A potential reason for this disconnect may be that most of the literature on the effect of pre-training on robustness uses models trained on large datasets, such as ImageNet [36], to generate pre-trained weights. Our method, in contrast, re-uses the same data for both pre-training and classification (Section 2.8 and Section 2.9). Since the extensive variation in a large training set often leads to models that are more resistant to perturbations in input data [27], it might be the case that models pre-trained on ImageNet’s 14 million images are more robust simply due to the nature of the dataset size.

Despite the small dataset size used to generate our pre-trained weights, the max-vote accuracy improvements that we presented in all tasks in the non-perturbed case support the notion that large-scale datasets are not necessary for pre-training; even unsupervised pre-training on small-scale datasets can provide a boost in performance [25,37].

### 3.4. Similarities between Learned Representations

Table 5 and Table 6, respectively, show the CKA similarity for representations from the first and last XceptionTime modules between models with different training sets ($Trn_{PD}$, $Trn_{PD}\cup Trn_{HOA}$) and with different weight initializations (RandInit, $W_{PD}$, $W_{PD,HOA}$). Overall, we can observe a dissimilarity between the representations from models with different attributes, supporting the idea that the choice of training data and weight initialization for a model has a significant impact on its feature space [26,38]. We do note some exceptions to this, namely for the aggregate case. The similarities between representations from the last XceptionTime module seem to be consistently higher than those from other individual tasks. One possible explanation is that since filtering motion sequences for specific tasks is not executed in the aggregate case, the models are trained on more data, which can lead to similar feature spaces. However, more research on this is required to give a more conclusive explanation.

Another important finding is that the feature similarities between models initialized with $W_{PD}$ and $W_{PD,HOA}$ are higher than the similarities between the same models and those using RandInit for the majority of the cases. This corroborates the findings in [38], highlighting the role of feature re-use when using pre-trained weights, a phenomenon not seen with random initialization.

### 3.5. Performance Differences among Tasks

Consistent with prior work [24], we found hand movement tasks to yield the best overall classification performance, considering both the segmented and max-vote accuracy (see Table 2 and Table 3). Using segmented and max-vote data, the best overall classification performance was achieved using the hand movement task, consistent with the identification of PD-related motor changes during virtual reach-to-grasp movements [39]. However, the greatest improvement from the baseline segmented performance was in finger tapping tasks, which is consistent with the use of kinematic data from finger tapping in prior efforts to classify PD-related motor changes [40].

Table 4 shows that, among all tasks, the max-vote accuracy increased the most when adding HOA training data for the pronation–supination hand task (about 6% improvement). This finding may be due to the fact that patients who claim to be healthy often actually have limited pronation in the forearm, since pronation can be recompensed by shoulder abduction and elbow flexion [41]. As a result, motion data from HOA may be characteristically more similar to motion data from PD patients for pronation–supination compared to other tasks. Thus, future studies with healthy young adults as participants would help in providing a benchmark for both aging and PD-related changes in upper extremity functions.

Furthermore, while most tasks demonstrated a considerable improvement over baseline models, toe tapping tasks did not demonstrate an improvement in performance. These findings may be partly due to the dissimilarity of kinematics in healthy controls relative to PD participants while toe tapping, but necessitate further investigation. The current findings are consistent with the prior findings of a good reliability of bradykinesia evaluations using individual upper extremity movements [42] and bradykinesia evaluations using hand grasping tasks [43].

## 4. Conclusions

In this study, we find that expanding training datasets with HOA motion data using unsupervised learning and transferring pre-trained weights for initialization can improve the motor impairment classification performance of a continuous motion data monitor for PD patients in telemedicine applications. Our method shows particular promise in low-data domains as it does not use additional PD or annotated data compared to the baseline classification model. Thus, the incorporation of data from healthy individuals could be instrumental in refining deep learning classification models tailored for neurological disorders, serving as both a baseline and a point of reference. Such an approach could pave the way for the development of extensive datasets, since acquiring data from healthy individuals is more straightforward and may provide an easier way to enhance models in future work.

In particular, we find that the addition of HOA training data improves both the classification accuracy and the robustness for most tasks in a clinical motor impairment evaluation. Furthermore, we show that unsupervised pre-training, even on small scale datasets, can provide a boost in motor impairment classification performance. Our observation that CKA feature similarities are generally higher between pre-trained classifiers than between a pre-trained and randomly initialized classifier suggests that pre-trained models exhibit feature re-use.

While these result are promising, future work should focus on evaluating the current approach in a larger cohort by either collecting more PD and HOA training data, collecting healthy young adult data to establish baseline age-related changes, or by generating synthetic data with generative machine learning models. Additionally, a natural expansion of this work is to consider methods to expand the feature selection of the input data, for instance, by using data from additional IMU sensors placed on different parts of the upper or lower extremities, using spectral components of IMU motion data (with fast Fourier transforms or spectrograms), or using multiple orders of differentiation of IMU motion data. A multi-modal approach should also be considered, which uses data from different domains such as muscle activations (retrieved from electromyogram sensors).

## Figures and Tables

**Table 1:** A summary of the attributes of each of the 10 supervised XceptionTime models we trained. Abbreviations: FTA = fine-tune all; FTL = fine-tune last, then all; HOA = healthy older adult; PD = Parkinson’s disease; RandInit = random initialization; *W* = weight.

Training Dataset	Weight Initialization	Fine-Tuning Scheme
$Trn_{PD}$	RandInit	-
$Trn_{PD}$	$W_{PD}$	FTL
$Trn_{PD}$	$W_{PD}$	FTA
$Trn_{PD}$	$W_{PD,HOA}$	FTL
$Trn_{PD}$	$W_{PD,HOA}$	FTA
$Trn_{PD}\cup Trn_{HOA}$	RandInit	-
$Trn_{PD}\cup Trn_{HOA}$	$W_{PD}$	FTL
$Trn_{PD}\cup Trn_{HOA}$	$W_{PD}$	FTA
$Trn_{PD}\cup Trn_{HOA}$	$W_{PD,HOA}$	FTL
$Trn_{PD}\cup Trn_{HOA}$	$W_{PD,HOA}$	FTA

**Table 2:** The models with the best segmented accuracy (and F1 score as tie-breaker) in each task. The best accuracy and best accuracy increases from baseline have been highlighted in bold. Abbreviations: FTA = fine-tune all; FTL = fine-tune last, then all; FT = finger tapping; HM = hand movements; HOA = healthy older adult; LA = leg agility; PD = Parkinson’s disease; PS = pronation–supination; TT = toe tapping; *W* = weights.

Task	Baseline Model			Best Model		
	Avg. Seg. Acc.	Train Set	Weight Init.	Fine-Tuning	Avg. Seg. Acc.	Δ from Baseline
PS	0.612 ± 0.087	$Trn_{PD}\cup Trn_{HOA}$	$W_{PD}$	FTA	0.701 ± 0.066	0.089
HM	0.744 ± 0.059	$Trn_{PD}$	$W_{PD,HOA}$	FTA	**0.789 ± 0.072**	0.045
FT	0.586 ± 0.096	$Trn_{PD}\cup Trn_{HOA}$	$W_{PD}$	FTL	0.680 ± 0.090	**0.094**
TT	0.693 ± 0.115	$Trn_{PD}$	$W_{PD}$	FTA	0.728 ± 0.098	0.035
LA	0.695 ± 0.077	$Trn_{PD}$	$W_{PD,HOA}$	FTL	0.773 ± 0.134	0.078
Agg.	0.745 ± 0.069	$Trn_{PD}$	$W_{PD}$	FTA	0.760 ± 0.016	0.015

**Table 3:** The models with the best max-vote accuracy (and F1 score as tie-breaker) on each task. The best accuracy and best accuracy increases from baseline have been highlighted in bold. Abbreviations: FTA = fine-tune all; FTL = fine-tune last, then all; FT = finger tapping; HM = hand movements; HOA = healthy older adult; LA = leg agility; MV = max-vote; PD = Parkinson’s disease; PS = pronation–supination; RandInit = random initialization; TT = toe tapping; *W* = weights.

Task	Baseline Model			Best Model		
	Avg. MV Acc.	Train Set	Weight Init.	Fine-Tuning	Avg. MV Acc.	Δ from Baseline
PS	0.594 ± 0.197	$Trn_{PD}\cup Trn_{HOA}$	$W_{PD}$	FTA	0.694 ± 0.121	0.100
HM	0.798 ± 0.087	$Trn_{PD}\cup Trn_{HOA}$	$W_{PD}$	FTL	**0.920 ± 0.075**	**0.122**
FT	0.647 ± 0.098	$Trn_{PD}\cup Trn_{HOA}$	RandInit	-	0.713 ± 0.142	0.066
TT	0.775 ± 0.122	$Trn_{PD}$	$W_{PD}$	FTA	0.775 ± 0.122	0.000
LA	0.756 ± 0.046	$Trn_{PD}\cup Trn_{HOA}$	$W_{PD}$	FTL	0.860 ± 0.080	0.104
Agg.	0.777 ± 0.047	$Trn_{PD}\cup Trn_{HOA}$	RandInit	-	0.812 ± 0.036	0.035

**Table 4:** The average accuracy of models, grouped by attribute, on test sets for each task. Performance metrics on both test sets with and without Gaussian noise added are recorded. Bold indicates the best max-vote accuracy among the different choices for each model attribute. Abbreviations: FT = finger tapping; HM = hand movements; HOA = healthy older adult; LA = leg agility; MV = max-vote; PD = Parkinson’s disease; PS = pronation–supination; RandInit = random initialization; TT = toe tapping; *W* = weights.

Task	Model Attributes		Without Gaussian Noise		With Gaussian Noise	
			Avg. Seg. Acc.	Avg. MV Acc.	Avg. Seg. Acc.	Avg. MV Acc.
PS	Train Set	$Trn_{PD}$	0.602 ± 0.097	0.603 ± 0.148	0.596 ± 0.078	0.518 ± 0.093
		$Trn_{PD}\cup Trn_{HOA}$	0.649 ± 0.097	**0.662 ± 0.113**	0.606 ± 0.090	**0.556 ± 0.087**
	Weight Init	RandInit	0.631 ± 0.084	0.632 ± 0.162	0.619 ± 0.063	**0.544 ± 0.069**
		$W_{PD}$	0.628 ± 0.088	**0.642 ± 0.127**	0.599 ± 0.085	**0.544 ± 0.069**
		$W_{PD,HOA}$	0.620 ± 0.116	0.624 ± 0.126	0.594 ± 0.091	0.526 ± 0.117
HM	Train Set	$Trn_{PD}$	0.753 ± 0.062	**0.837 ± 0.077**	0.631 ± 0.088	0.655 ± 0.133
		$Trn_{PD}\cup Trn_{HOA}$	0.748 ± 0.069	0.832 ± 0.090	0.649 ± 0.086	**0.702 ± 0.097**
	Weight Init	RandInit	0.739 ± 0.066	0.796 ± 0.092	0.624 ± 0.106	0.636 ± 0.176
		$W_{PD}$	0.756 ± 0.054	**0.852 ± 0.078**	0.642 ± 0.081	0.683 ± 0.109
		$W_{PD,HOA}$	0.751 ± 0.075	0.837 ± 0.077	0.645 ± 0.082	**0.695 ± 0.08**
FT	Train Set	$Trn_{PD}$	0.612 ± 0.102	0.668 ± 0.111	0.538 ± 0.104	0.587 ± 0.145
		$Trn_{PD}\cup Trn_{HOA}$	0.633 ± 0.086	**0.690 ± 0.093**	0.565 ± 0.086	**0.640 ± 0.099**
	Weight Init	RandInit	0.598 ± 0.093	0.680 ± 0.126	0.527 ± 0.107	0.573 ± 0.175
		$W_{PD}$	0.636 ± 0.095	**0.698 ± 0.088**	0.539 ± 0.091	0.614 ± 0.109
		$W_{PD,HOA}$	0.622 ± 0.093	0.658 ± 0.101	0.576 ± 0.091	**0.632 ± 0.109**
TT	Train Set	$Trn_{PD}$	0.693 ± 0.112	**0.775 ± 0.106**	0.554 ± 0.109	**0.450 ± 0.071**
		$Trn_{PD}\cup Trn_{HOA}$	0.619 ± 0.125	0.715 ± 0.182	0.523 ± 0.130	0.415 ± 0.126
	Weight Init	RandInit	0.673 ± 0.112	0.738 ± 0.131	0.565 ± 0.097	**0.450 ± 0.061**
		$W_{PD}$	0.652 ± 0.132	**0.756 ± 0.165**	0.523 ± 0.130	0.415 ± 0.126
		$W_{PD,HOA}$	0.652 ± 0.123	0.738 ± 0.147	0.541 ± 0.123	0.438 ± 0.093
LA	Train Set	$Trn_{PD}$	0.732 ± 0.104	0.810 ± 0.0819	0.726 ± 0.119	0.798 ± 0.066
		$Trn_{PD}\cup Trn_{HOA}$	0.746 ± 0.093	**0.830 ± 0.067**	0.750 ± 0.101	**0.806 ± 0.052**
	Weight Init	RandInit	0.715 ± 0.089	0.777 ± 0.057	0.739 ± 0.116	0.798 ± 0.06
		$W_{PD}$	0.735 ± 0.104	0.823 ± 0.075	0.731 ± 0.109	0.798 ± 0.060
		$W_{PD,HOA}$	0.755 ± 0.095	**0.838 ± 0.074**	0.744 ± 0.109	**0.808 ± 0.059**
Agg.	Train Set	$Trn_{PD}$	0.752 ± 0.035	0.783 ± 0.039	0.588 ± 0.052	0.532 ± 0.097
		$Trn_{PD}\cup Trn_{HOA}$	0.753 ± 0.047	**0.793 ± 0.062**	0.603 ± 0.046	**0.563 ± 0.089**
	Weight Init	RandInit	0.748 ± 0.058	0.794 ± 0.045	0.613 ± 0.058	**0.585 ± 0.106**
		$W_{PD}$	0.753 ± 0.030	**0.786 ± 0.05**	0.609 ± 0.037	0.564 ± 0.083
		$W_{PD,HOA}$	0.753 ± 0.042	**0.786 ± 0.058**	0.573 ± 0.047	0.513 ± 0.086

**Table 5:** Similarities between the representations from the first and last XceptionTime module for each supervised model, grouped by training set. Abbreviations: CKA = centered kernel alignment; FT = finger tapping; HM = hand movements; HOA = healthy older adult; LA = leg agility; PD = Parkinson’s disease; PS = pronation–supination; TT = toe tapping.

Module	Train Sets to Compare	CKA Similarity					
		PS	HM	FT	TT	LA	Agg.
First	$Trn_{PD}$/$Trn_{PD}\cup Trn_{HOA}$	0.406	0.451	0.344	0.481	0.554	0.338
Last	$Trn_{PD}$/$Trn_{PD}\cup Trn_{HOA}$	0.456	0.158	0.460	0.650	0.431	0.823

**Table 6:** Similarities between the representations from the first and last XceptionTime module for each supervised model, grouped by weight initialization. Abbreviations: CKA = centered kernel alignment; FT = finger tapping; HM = hand movements; HOA = healthy older adult; LA = leg agility; PD = Parkinson’s disease; PS = pronation–supination; RandInit = random initialization; TT = toe tapping; *W* = weights.

Module	Inits. to Compare	CKA Similarity					
		PS	HM	FT	TT	LA	Agg.
First	RandInit/$W_{PD}$	0.134	0.118	0.125	0.221	0.090	0.143
	RandInit/$W_{PD,HOA}$	0.099	0.177	0.291	0.063	0.090	0.097
	$W_{PD}$/$W_{PD,HOA}$	0.558	0.209	0.482	0.233	0.584	0.225
Last	RandInit/$W_{PD}$	0.123	0.437	0.371	0.305	0.344	0.855
	RandInit/$W_{PD,HOA}$	0.151	0.102	0.484	0.307	0.475	0.629
	$W_{PD}$/$W_{PD,HOA}$	0.245	0.168	0.453	0.327	0.808	0.842

## Data Availability

Data used for analysis are available in an open-source dataset [19].

## Abbreviations

The following abbreviations are used in this manuscript:

CKA	centered kernel alignment
FTA	fine-tune all
FTL	fine-tune last, then all
FT	finger tapping
HM	hand movement
HOA	healthy older adult
LA	leg agility
MV	max-vote
PD	Parkinson’s disease
PS	pronation–supination
RandInit	random initialization
TT	toe tapping
*W*	weights
